# Pediatric Oral Mucocele Management: A Case Series Investigating Different Treatment Approaches

**DOI:** 10.7759/cureus.63342

**Published:** 2024-06-28

**Authors:** Divya Mukundan, Ramesh R

**Affiliations:** 1 Pediatric and Preventive Dentistry, Saveetha Dental College and Hospitals, Saveetha Institute of Medical and Technical Sciences, Saveetha University, Chennai, IND

**Keywords:** electrocautery, scalpel, mucocele, lower lip, laser

## Abstract

A mucocele is a benign cystic lesion containing clear fluid within a thin capsule, typically resulting from the disruption of minor salivary glands and leakage into surrounding tissues, most commonly on the lower lip. These lesions often arise due to traumatic injuries such as lip-sucking, biting, or trauma from orthodontic appliances. This study compares different surgical methods for mucocele removal in pediatric patients through three distinct cases.

This study includes three pediatric cases of mucocele removal using different surgical methods. Case 1 involved a nine-year-old girl with a traumatic bite on the lower lip, treated with a traditional approach using a scalpel. The surgical excision resulted in adequate healing with no recurrence after six months. Case 2 described a 12-year-old boy with a lip-biting habit, who presented with a swelling on the lower left lip. He underwent diode laser treatment, which facilitated faster healing, minimal discomfort, and no scarring after 30 days. Case 3 involved a 14-year-old boy, also with a lip-biting habit, who had swelling on the lower left lip. Electrocautery was used for his treatment, resulting in minimal bleeding, effective healing after 21 days, and no recurrence at the six-month follow-up. These cases demonstrate the efficacy of different treatment modalities for mucocele removal in pediatric patients.

Evaluations of pain, bleeding, and swelling indicated that minimally invasive methods like the diode laser offer significant benefits in patient comfort and recovery. These findings highlight the potential of minimally invasive techniques to enhance the management of mucoceles in pediatric patients, underscoring the need for further research to determine the long-term efficacy of various treatment modalities.

## Introduction

Mucoceles are the most prevalent oral lesion, occurring in about 2.4 out of every 1,000 individuals [1]. The term mucocele is derived from the word “mouco” meaning mucus and “coele” meaning cavity [2]. Mucoceles often occur in the second decade of life and are uncommon in children under one year of age [3]. Mucocele can manifest as either an extravasation or retention cyst [4]. A ruptured salivary gland duct and the ensuing leakage into the surrounding soft tissues cause extravasation cysts. When the salivary gland ducts are blocked, glandular production is reduced or absent, which results in a retention cyst [4]. Numerous investigations indicate that there is no clinical distinction between extravasation and retention of mucocele based on gender [5]. The most common type is extravasation cyst, where mucous pools into and surrounds granulation tissue to generate mucous extravasation cysts, which make up about 92% of cases. Retention cysts in the epithelium lining make up the remaining 1%, or about 8% [6]. Although the frequency of mucoceles in children under one year is unknown, it is more common in younger people than in adults [3].

With recent developments in pediatric dentistry, it is of utmost importance to carefully choose the course of treatment for mucocele. The surgical extraction of the mucocele typically results in unavoidable postoperative discomfort for children and young adolescents [7]. Coming up with a treatment strategy that causes the least amount of discomfort is essential, particularly when treating young children, as it affects their cognitive development and behavior guidance in the future. Mucoceles can be treated with invasive or non-invasive methods. Most skilled dentists can do surgical excision, which has minimal cost and minimal equipment requirements. The most common recommended treatment for the lesion previously was surgical excision, but inadequate excision may lead to more frequent recurrence [8]. Laser ablation (CO_2_, Er, Cr: YSGG), electrosurgery, cryosurgery, micro-marsupialization, and intralesional steroids are non-invasive methods for treating mucocele [9]. Laser treatment is well-received in children due to its non-invasiveness and quicker recovery time, while surgical excision is preferred for cases requiring complete removal of the affected area. Patients highly favor laser and electrosurgical procedures due to the minimal use of local anesthesia, low levels of dental fear and anxiety, minimal discomfort, absence of bleeding, and minimal scarring during the healing process [10]. Electrosurgery is similar to lasers in terms of mode of use and patient acceptance. Treating mucocele in children should be carefully planned considering the patient's age, level of cooperation, and lesion size, taking into account the recurrence rates associated with each treatment option. The aim of the article is to highlight the first-of-its-kind case series to comprehensively compare all three commonly used modalities used in the management of mucocele on the lower lip treated by surgical excision, electrocautery, and diode lasers.

## Case presentation

Case 1

A nine-year-old female patient reported to the Outpatient Department of Pediatric and Preventive Dentistry at Saveetha Dental College and Hospitals, Chennai, with a chief complaint of swelling on the lower lip for four weeks. The patient was asymptomatic with no significant contributing medical, dental, or drug history, along with family history. It was noted that the patient had a traumatic bite on the lower labial mucosa a few months ago.

On extraoral examination, the face was bilaterally symmetrical with competent lips. Lymph nodes were not palpable. Based on inspection, the swelling was well-circumscribed, measuring 1x1 cm, with normal-appearing mucosa and a slightly bluish hue near teeth 41 and 42. Upon palpation, the swelling was fluctuant and sessile with no associated pain. There was no difficulty in chewing or speaking. No other anomalies were detected. All routine blood tests, including a complete blood count (CBC), bleeding time, and clotting time, were within normal limits.

**Figure 1:**
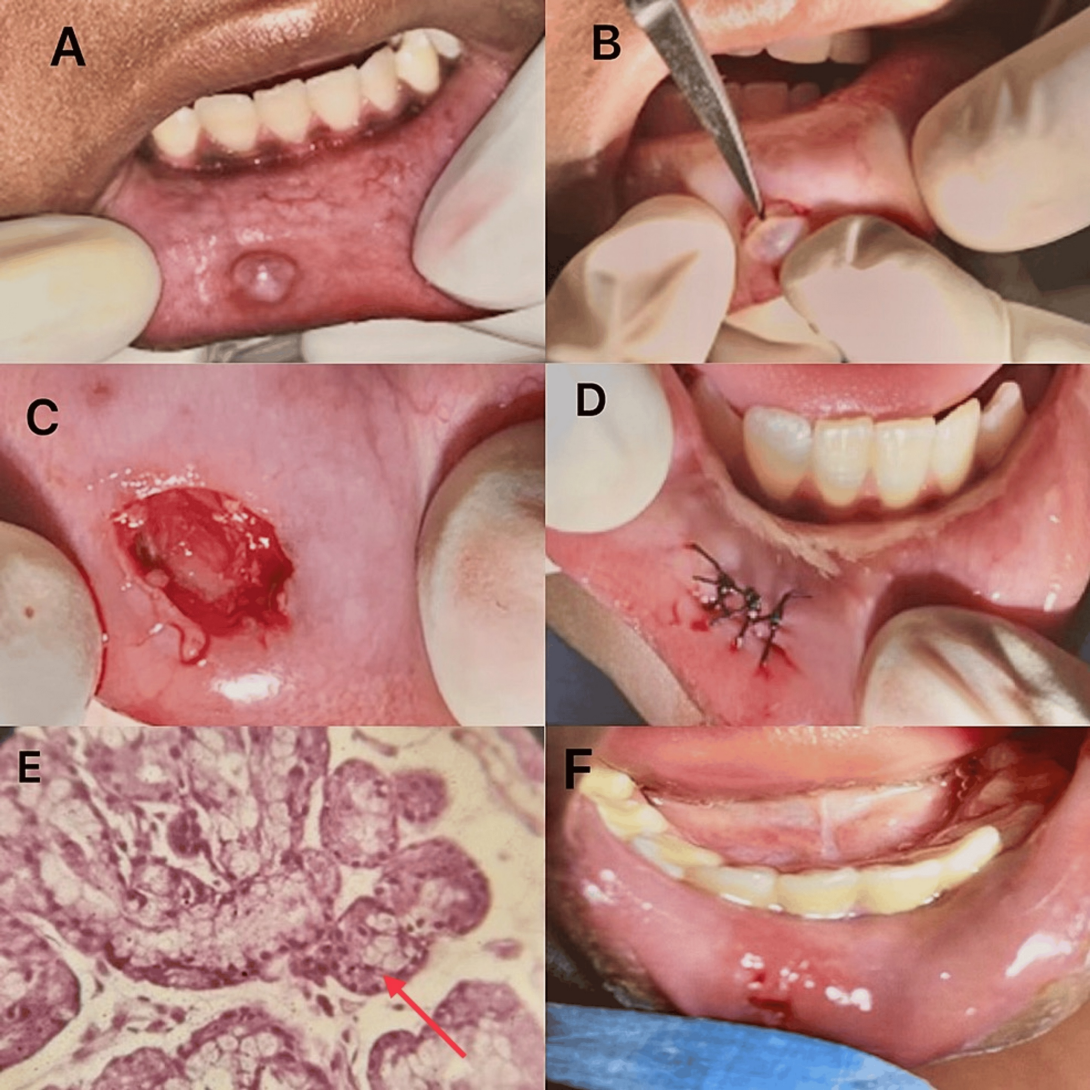
Excision of mucocele using scalpel (A) Swelling present in relation to 41 and 42 region; (B) Excision of the lesion using scalpel no. 15; (C) Immediate postoperative view; (D) Intermittent sutures placed; (E) Hematoxylin and eosin section displaying areas of mucin pooling encircled by granulation tissue in the center of the luminal area (indicated by a red arrow); (F) Postoperative healing after one week

Based on the history and clinical findings, a provisional diagnosis of mucocele was made. In this case, we used a scalpel for surgical excision. It is an inexpensive, practical form of treatment that can be easily carried out with adolescents. Surgical excision of the mucocele was done under local anesthesia of 2% lignocaine and 1:100,00 adrenaline (White Swan Pharmaceutical, India) after obtaining informed consent. The lesion was resected with scalpel no. 15 (Ribbel International Limited, India) from the base with the capsule intact. The excised tissue was preserved in a solution of 10% formalin and sent to the Department of Oral Pathology, where histopathological examination confirmed it was a mucocele, with hematoxylin and eosin sections displaying areas of mucin pooling surrounded by granulation tissue in the center of the luminal area. An intermittent suture was placed with 4-0 silk (Unisur Lifecare Pvt. Ltd., India), and postoperative instructions were given with the prescription of antibiotics and analgesia (C. Amoxicillin 250 mg and T. Paracetamol 250 mg). After seven days, the sutures were removed and mild scarring was present. At six-month follow-up, the healing was adequate, and there were no indications of a recurrence (Figure 1).

Case 2

A 12-year-old boy reported to the Outpatient Department of Pediatric and Preventive Dentistry at Saveetha Dental College and Hospital, Chennai, with a chief complaint of swelling in the lower left lip region for the past three weeks. There was no significant contributing medical, dental, and family history. The patient had a habit of lip biting, and this was the first time a swelling appeared that was initially small in size and attained the present size. On extraoral examination, no abnormalities were detected. Upon intraoral inspection, a well-circumscribed swelling measuring 1x1 cm with normal mucosal appearance was observed near teeth 32 and 33. Upon palpation, the swelling was found to be painless and sessile. No other abnormalities were noted.

**Figure 2:**
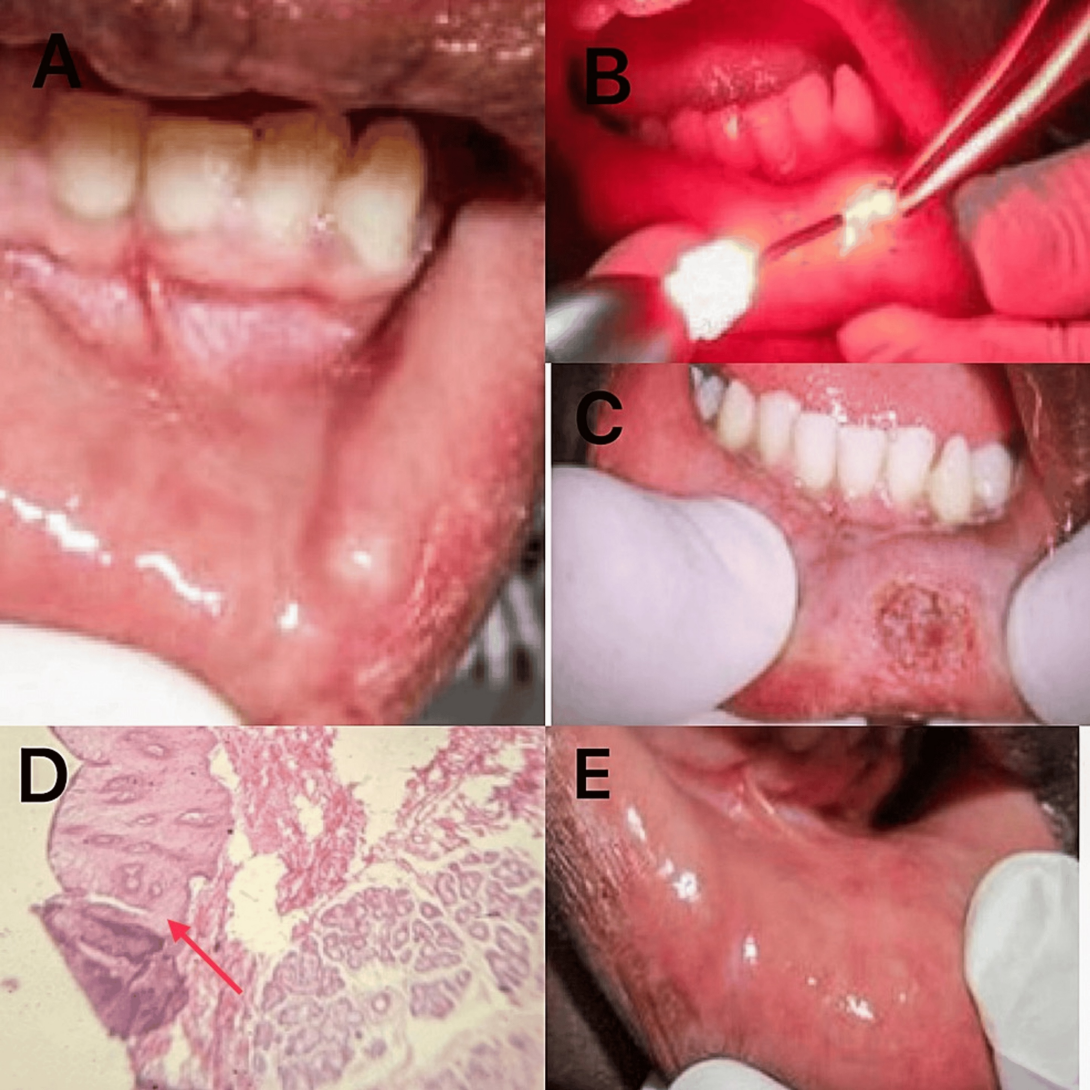
Mucocele excision using laser (A) Swelling present on the lower lip; (B) Laser beam being used to excise the lesion; (C) Immediate postoperative view; (D) Hematoxylin and eosin section displaying areas of mucin (indicated by a red arrow); (E) Clinical appearance after two weeks

Differential diagnoses included lipoma, fibroma, oral hemangioma, soft tissue abscess, oral lymphangioma, and benign salivary gland tumor. The provisional diagnosis of mucoceles was made based on its rapid onset, bluish hue, and fluctuation observed during the clinical examination, as well as the patient's history of trauma. Laser excision was explained to the patient and parent. Informed consent was obtained to perform the most recent treatment option of the laser. Excision of mucocele was done under local anesthesia with 2% lignocaine and 1:100,00 adrenaline. The lesion was excised using a soft tissue laser (diode laser; Biolase, United States), 840 nm in wavelength, 4µ diameter tip at three watts in continuous mode. The excised tissue was preserved in a solution of 10% formalin and sent to the Department of Oral Pathology, where it was analyzed histopathologically and identified as a mucocele, with hematoxylin and eosin sections showing mucin pools encircled by granulation tissue within the luminal center. Analgesics (T. Paracetamol 250mg) were administered, and the patient was advised to take them in case of pain. There was uneventful healing with minimal patient discomfort. The patient was instructed to discontinue the practice of biting their lips. At six-month follow-up, there was no scarring (Figure 2). 

Case 3

A 14-year-old male reported to the Outpatient Department of Pediatric and Preventive Dentistry at Saveetha Dental College and Hospital, Chennai, with a chief complaint of swelling in the lower left lip region for one month. There was no significant medical, dental, drug, or family history. The patient had a habit of lip-biting. On extraoral examination, no abnormalities were detected. On intraoral inspection, a 1.5x1.5 cm well-circumscribed swelling was observed near the 42 and 43 regions. Upon palpation, the swelling was fluctuant and sessile with no associated pain.

**Figure 3:**
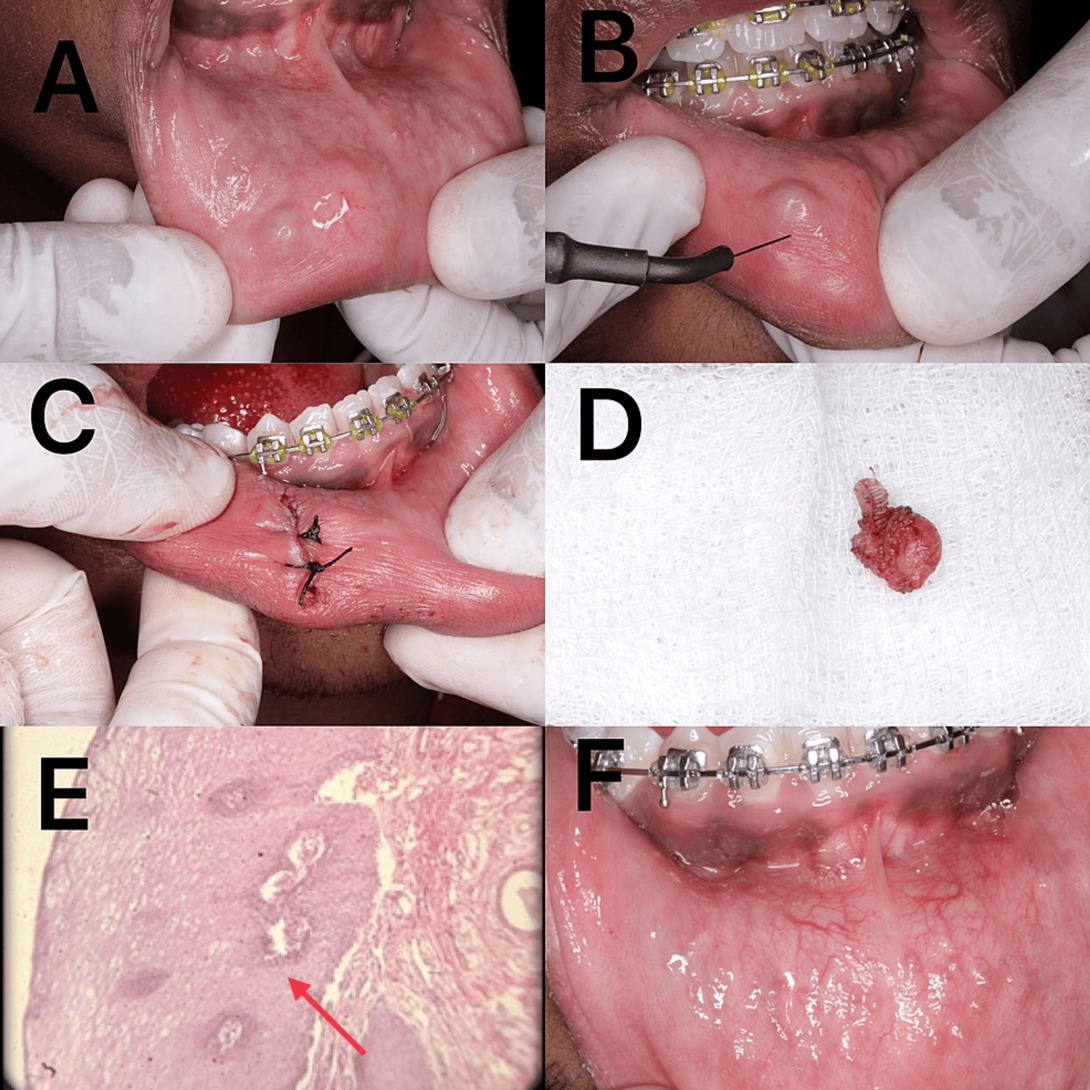
Mucocele excision using electrocautery (A) Swelling present on the lower lip; (B) Electrocautery being used to excise the lesion; (C) Sutures placed after excision of the lesion; (D) Tissue excised using electrocautery; (E) Histopathological appearance (indicated by a red arrow); (F) Clinical appearance after two weeks

The swelling was initially small and attained the present size. The swelling was painless and sessile. No other abnormalities were present. On the basis of the history and clinical examination, a provisional diagnosis of mucocele was made. The treatment plan involved a comprehensive surgical procedure using electrocautery to remove the affected area completely. After obtaining informed consent from the patient and explaining the procedure to both the patient and their parents, the procedure was carried out under local anesthesia of 2% lignocaine and 1:100,00 adrenaline. The electrocautery (PerFect TCS II, Coltene, United States) was set to 'cut + coagulant 1' mode with a speed of five and a T2 tip to remove the mucocele. Prior to excision, grounding was achieved by momentarily placing the electrode tip in a charcoal holder to ensure optimal conductivity. The patient fully cooperated throughout the procedure. The most effective method for facilitating minimally invasive treatment of the lesion was through circular motion around its periphery. Excision of minor salivary glands along with the duct was performed meticulously surrounding the lesion to prevent recurrence. The excised tissue was preserved in a 10% formalin solution before being transferred to the Department of Oral Pathology for histological analysis. There, it was determined to be a mucocele; the hematoxylin and eosin section revealed mucin pools surrounded by granulation tissue within the luminal core. The patient was advised to refrain from biting their lips and consuming spicy food as part of the postoperative guidelines. There was minimal bleeding during and after the procedure, however, an intermittent suture was placed with 4-0 silk to promote better healing. The patient was administered mucopain gel to alleviate postoperative discomfort. Mucopain gel contains 20% benzocaine (ICPA Health Products Ltd., India). The healing stage was monitored seven days following the excision of the mucocele, and no complaints were reported. At six-month follow-up, no scarring and no recurrence was observed (Figure 3).

An assessment of pain, bleeding, and swelling was conducted for all the techniques (Table 1).

**Table 1:** A comparative analysis of scalpel, laser, and electrocautery VAS: Visual Analogue Scale

Parameters	Excision With Scalpel	Excision With Laser	Excision With Electrocautery
History	Trauma due to biting	Lip-biting habit	Lip-biting habit
Bleeding assessment	Controlled after suturing	No bleeding	Minimal bleeding
Sutures	Sutures placed with 4-0 silk	No sutures placed	Sutures placed with 4-0 silk
Pain evaluation by VAS scale	Moderate pain was reported until the fifth day	Mild pain was reported only for one day	Mild pain was reported until the third day
Analgesics taken (T. Para 250 mg)	Taken for three days	No analgesics taken	Taken only once after the procedure
Postoperative healing	Sutures were removed on the seventh with no scarring	Healing was satisfactory with no scaring	Sutures were removed on the seventh with healing satisfactory and no scarring
Follow-up	After six months, no recurrence	After six months, no recurrence	After six months, no recurrence

## Discussion

Mucocele is the second most common oral lesion commonly seen, and the lower lip is the most affected area, followed by the tongue, floor of the mouth, and buccal mucosa [11]. The lips consist of adipose tissue, connective tissue, blood vessels, nerves, and salivary glands. Therefore, any abnormality in these tissues might result in swelling of the lips. Mucocele, fibroma, lipoma, mucus retention cyst, sialolith, phlebolith, and salivary gland neoplasm manifest as lip swelling. Nevertheless, they can be differentiated from mucocele by their clinical presentation, color, texture, cause, and site of occurrence. Research by Bagán et al. [12], on 25 patients with mucoceles revealed that while 48% identified their lesions visually despite being asymptomatic, another 48% had their lesions discovered incidentally by a specialist, and only 4% experienced vague discomfort without pain. Small salivary gland mucoceles are always superficial, rarely exceeding 1.5 cm in diameter, and can cause convex swelling that may hinder chewing or speaking, depending on their size and location [13]. Mucoceles can be treated using a variety of techniques, like scalpel, laser, electrocautery, or marsupialization [14,15]. In the present research, mucoceles were treated using a scalpel, laser, and electrocautery. 

Many cases recommend surgical excision as the preferred therapy as it is a cost-effective procedure that does not require sophisticated equipment and can be conducted by most experienced dentists. This treatment involves removing the affected accessory salivary gland. Elliptical incisions are the most frequently used incision method. This helps to reduce the level of mucosal tissue loss, the incidence of developing fibrous scars, and the leakage of cystic fluid, which may be responsible for recurrence [16]. A limitation of this approach is that it necessitates precise manipulation of the instrument with a high level of tactile sensitivity. The likelihood of experiencing postoperative bleeding is greater compared to alternative treatment methods like laser therapy. Additionally, there is a greater probability of developing a more ulcerative look and maybe experiencing a longer healing period [17,18].

Foroughiasl et al. [19] reported that when an electrocautery was used instead of a laser to remove a mucocele, the healing process took longer and there was more postoperative bleeding. The laser is a very precise device for removing tissue that has certain benefits in comparison to the scalpel. The laser induces minimal damage to the surrounding tissues, particularly the underlying layers of muscle [20]. The described scenario results in low postoperative bleeding, which can be attributed to the coagulation capabilities of the laser. Postoperative recovery was faster, and scar formation was minimized due to the limited damage to the surrounding tissues.

The diode laser has gained significance in the field of dentistry. The diode laser, with a wavelength of 800-810 nm, is strongly absorbed by hemoglobin. This absorption leads to an increase in temperature and facilitates the coagulation and carbonization of soft tissues, namely the oral mucosa. The benefits of using a diode laser for mucocele excision include a surgical field without bleeding, minimal discomfort, minimal scarring, and much reduced or no pain after the procedure. In order to minimize unfavorable postoperative symptoms and severe heat injury to soft tissues, the power level should be adjusted correctly [2].

While numerous case series have compared scalpel and laser techniques for mucocele removal [21,22], this study stands out as the first of its kind to comprehensively compare all three commonly used procedures for this purpose. When compared to surgical and electrosurgical excision, the laser resection in this case series showed less postoperative discomfort and better clinical healing. In order to completely eradicate the inclination to bite one's lips, postoperative instructions are crucial once the affected area has been removed. Moreover, it is necessary to implement educational programs to raise awareness among parents and kids about the need to stop lip-biting behavior. If a child's long-standing innate fear is the reason behind the habit's persistence, it is imperative to look into the underlying causes and seek professional psychological help [22,23].

## Conclusions

In conclusion, the management of mucoceles in pediatric patients can be effectively achieved through various surgical methods, each offering distinct advantages. Traditional scalpel excision, as shown in the first case, is effective with satisfactory healing. Minimally invasive techniques like diode laser and electrocautery, demonstrated in the second and third cases, provide faster healing, reduced postoperative discomfort, minimal scarring, and lower recurrence rates. These findings highlight that diode laser treatment may be a better choice for improving patient comfort and recovery. Further research is needed to evaluate the long-term efficacy and comparative effectiveness of these modalities in a larger pediatric population. Adopting minimally invasive techniques promises better clinical outcomes and quality of life for young patients.
